# Molecular Mechanism and Application of Somatic Cell Cloning in Mammals—Past, Present and Future

**DOI:** 10.3390/ijms232213786

**Published:** 2022-11-09

**Authors:** Marcin Samiec

**Affiliations:** Department of Reproductive Biotechnology and Cryoconservation, National Research Institute of Animal Production, Krakowska 1 Street, 32-083 Balice, Poland; marcin.samiec@iz.edu.pl

Thus far, nearly 25 mammalian species have been cloned by intra- or interspecies somatic cell nuclear transfer (SCNT). Among them, non-transgenic and transgenic representatives of such domesticated and wild-living animals that have been propagated and/or multiplied by intraspecific or interspecific SCNT-based cloning are:

- Pigs (*Sus scrofa domesticus*) [1,2];
- Sheep (*Ovis aries*) [3,4];
- Goats (*Capra aegagrus hircus*) [5,6];
- Cattle (*Bos taurus taurus*) [7,8,9];
- Horses (*Equus ferus caballus*) [10];
- Mules (equine hybrids: *Equus asinus × Equus ferus caballus*) [11];
- Dromedary camels (*Camelus dromedarius*) [12];
- Bactrian camels (*Camelus bactrianus ferus*) [13];
- Water buffalos (*Bubalus bubalis*) [14];
- Rabbits (*Oryctolagus cuniculus*) [15];
- Domestic cats (*Felis silvestris catus*) [16];
- Domestic dogs (*Canis lupus familiaris*) [17];
- Mice (*Mus musculus musculus*) [18];
- Rats (*Rattus norvegicus domestica*) [19];
- Ferrets (*Mustela putorius furo*) [20];
- Mouflon (*Ovis aries/ammon musimon*) [21];
- Gaur (*Bos gaurus*) [22];
- Red deer (*Cervus elaphus*) [23];
- Pyrenean ibex (bucardo; *Capra pyrenaica pyrenaica*) [24];
- African wild cat (*Felis silvestris lybica*) [25];
- Sand cat (*Felis margarita margarita*) [26];
- Gray wolf (*Canis lupus lupus*) [27];
- Coyote (*Canis latrans*) [28];
- Cynomolgus monkey/macaque (*Macaca fascicularis*) [29].

Despite the above-indicated abundant variety of SCNT-derived mammalian species, the effectiveness of SCNT-based cloning remains immensely or considerably low and oscillates between 0.1% and 5%, while estimating the outcomes of offspring born in relation to the total numbers of nuclear-transferred oocytes [30,31]. For this reason, at the present stage of investigations, extensive efforts are being undertaken to achieve considerable scientific breakthroughs, which would enable researchers to not only tremendously increase the ex vivo and in vivo developmental competences, but also to remarkably ameliorate the parameters related to the cytological, molecular and epigenetic qualities of SCNT-generated mammalian embryos. Only such a crucial turning point or a substantial research game changer would open up new possibilities for both improving the overall efficiency of SCNT-based cloning and, as a consequence, play an increasingly important role as an assisted reproductive technology (ART) which is characterized by a broad spectrum of applicability in embryology, biotechnology, transgenics and biomedicine [32,33].

It is beyond any doubt that the relatively or extremely low efficiency of mammalian SCNT-mediated cloning, including both its intra- and interspecies model, can only be improved by comprehensively recognizing molecular and epigenetic determinants and mechanisms affecting the developmental competences of SCNT-derived embryos [34]. A wide range of biological and molecular factors predestine and predominantly bias the biotechnological suitability of nuclear donor cells and nuclear recipient oocytes for SCNT-mediated ARTs. The extent of this suitability is measured and directly depends on the developmental capacity and quality parameters pinpointed for nuclear-transferred oocytes and corresponding somatic-cell-cloned embryos in different mammalian species [35]. The main impact on the development of cloned embryos is exerted by the type and provenance of nuclear donor cells [36,37,38]. In this context, an important role is played by the strategies used to artificially synchronize the mitotic cycle of nuclear donor cells expanded ex vivo at the G0/G1 stages [39,40]. Notably, the developmental outcomes of somatic-cell-cloned embryos are largely determined by the molecular quality parameters reflected in the incidence of apoptotic cell death and oxidative stress processes in the nuclear donor cells and SCNT-derived embryos cultured in vitro [40,41,42,43]. Additionally, it is worth highlighting that the developmental capability of cloned embryos is remarkably biased by the molecular quality of metaphase-II stage nuclear recipient oocytes, which largely depends on coordination between the processes of meiotic, cytoplasmic and epigenomic maturation [44,45,46]. Not without significance is the tremendous influence of the approaches applied to artificially activate the embryo-specific developmental program of SCNT-derived oocytes on the efficacy of propagating cloned embryos and their molecular quality [47,48,49]. Furthermore, the effectiveness of generating somatic-cell-cloned embryos results from the capabilities of donor cell nuclei to epigenetically reprogram their transcriptomic signatures in the cytoplasm of SCNT-derived oocytes and the blastomeres of corresponding cloned embryos [50,51]. In turn, the epigenomic reprogrammability of transcriptional activity within donor cell nuclei has been proven to be strongly affected by the molecular network of inter-relations between nuclear and mitochondrial genomes that has been established during the early embryonic development of activated SCNT-derived oocytes [52,53,54]. Finally, as a consequence of applying a wide variety of methods focused on modulating/transforming the transcriptional activities of donor cell nuclear genomes by extrinsic epigenetic modifiers such as non-selective inhibitors of histone deacetylases (HDACi) and/or non-selective inhibitors of DNA methyltransferases (DNMTi), the enhanced capabilities of donor cell nuclei to correctly and faithfully reprogram their transcriptomic profiles in SCNT-derived embryos have been shown [31,55,56].

In summary, this Special Issue will publish research articles and comprehensive review papers aimed at highlighting the state of the art and mechanistic insights into precisely identifying and unravelling a wide array of genomic, epigenomic, transcriptomic and proteomic factors which cumulatively determine the molecular parameters which are of paramount importance for the quality of nuclear donor cells, nuclear recipient oocytes and SCNT-derived embryos. Thoroughly deciphering the multifaceted nature of all the aforementioned factors and insightful interpretation of the biological crosstalk between them can finally bias the augmentation of the overall efficiency of SCNT-based cloning. This is a preponderant condition indispensable for the practical implementation of SCNT-mediated ARTs to various research fields and interdisciplinary studies at the interface of experimental and applied embryology, biotechnology, transgenics, biomedicine, biopharmacology, the creation of animal models for etiopathogenesis and physiopathology of human diseases and the genetic rescue and/or resurrection of endangered/extinct mammalian species.
